# Parallel Implementation of Katsevich's FBP Algorithm

**DOI:** 10.1155/IJBI/2006/17463

**Published:** 2006-05-03

**Authors:** Jiansheng Yang, Xiaohu Guo, Qiang Kong, Tie Zhou, Ming Jiang

**Affiliations:** LMAM, School of Mathematical Sciences, Peking University, Beijing 100871, China

## Abstract

For spiral cone-beam CT, parallel computing is an effective approach to resolving the problem of heavy computation burden. It is well known that the major computation time is spent in the backprojection step for either filtered-backprojection (FBP) or backprojected-filtration (BPF) algorithms. By the cone-beam cover method [1], the backprojection procedure is driven by cone-beam projections, and every cone-beam projection can be backprojected independently. Basing on this fact, we develop a parallel implementation of Katsevich's FBP algorithm. We do all the numerical experiments on a Linux cluster. In one typical experiment, the sequential reconstruction time is 781.3 seconds, while the parallel reconstruction time is 25.7 seconds with 32 processors.


Hindawi Publishing Corporation
International Journal of Biomedical Imaging
Volume 2006, Article ID 17463, Pages 1–8
DOI 10.1155/IJBI/2006/17463

### Parallel Implementation of Katsevich's FBP Algorithm

**Jiansheng Yang, Xiaohu Guo, Qiang Kong, Tie Zhou, and Ming Jiang**

*LMAM, School of Mathematical Sciences, Peking University, Beijing 100871, China*

Received 30 September 2005; Revised 17 January 2006; Accepted 17 February 2006

For spiral cone-beam CT, parallel computing is an effective approach to resolving the problem of heavy computation burden. It is well known that the major computation time is spent in the backprojection step for either filtered-backprojection (FBP) or backprojected-filtration (BPF) algorithms. By the cone-beam cover method [1], the backprojection procedure is driven by cone-beam projections, and every cone-beam projection can be backprojected independently. Basing on this fact, we develop a parallel implementation of Katsevich's FBP algorithm. We do all the numerical experiments on a Linux cluster. In one typical experiment, the sequential reconstruction time is 781.3 seconds, while the parallel reconstruction time is 25.7 seconds with 32 processors.

Copyright © 2006 Jiansheng Yang et al. This is an open access article distributed under the Creative Commons Attribution License, which permits unrestricted use, distribution, and reproduction in any medium, provided the original work is properly cited.

### 1. INTRODUCTION

Spiral cone-beam CT can be used for rapid volumetric imaging with high longitudinal resolution and for efficient utilization of X-ray source. Katsevich's filtered-backprojection (FBP) inversion formula represents a significant breakthrough in this area [2–4]. However, sequential implementation of this formula demands more intensive computation and hardware resources [2, 3]. Parallel computation technique provides an effective solution to this issue.

Parallel computation technique was previously applied to 2D CT. Nowinski [5] investigated four forms of parallelism in 2D CT: pixel, projection, ray, and operation parallelisms. Using the data-parallel programming style, Roerdink and Westenberg [6] studied parallel implementation for two standard 2D reconstruction algorithms: FBP and direct Fourier reconstruction. There were also some results on parallel implementations of 3D CT image reconstructions for parallel-beam and fan-beam geometries [7, 8]. For cone-beam CT, parallel implementations of the Feldkamp algorithm on Beowulf clusters were reported, typically based on smart communication schemes [9, 10]. In [9], a master node does all the weighting and filtering of the projections, while the other nodes perform the backprojection for their assigned image subvolumes, respectively. In [10], each node weights and filters the projection data assigned to it, and then accomplishes the backprojection for its assigned image subvolume in a send-receive mode.

For image reconstruction from cone-beam projections, the backprojection step is extremely time-consuming. In [1], the authors proposed the cone-beam cover method for the backprojection. This method is different from the conventional methods based on PI line, and provides an alternative efficient implementation scheme for Katsevich's FBP formula and its kind. In the cone-beam cover method, any filtered cone-beam projection can be backprojected independently. On the ground of this independence, we present a parallel implementation for Katsevich's exact reconstruction formula in this manuscript. Numerical simulations demonstrate the high performance of the proposed parallel scheme with remarkable runtime reduction. For the 3D Shepp-Logan phantom [11] with $256 \times 256 \times 256$ voxels, $3 \times 600$ source points (600 points per turn) and $100 \times 500$ cone-beam projection at every source point, the sequential cone-beam cover algorithm takes 781.3 seconds to reconstruct the volume image, while the proposed parallel implementation only needs 25.7 seconds with 32 processors on the same Linux cluster.

The structure of this manuscript is as follows. Section 2 introduces briefly Katsevich's inversion formula and the cone-beam cover method. Section 3 describes the proposed parallel implementation of Katsevich's inversion formula. Section 4 provides experiment results and details of the computing environment. Section 5 discusses relevant issues. Finally, Section 6 concludes the paper.

### 2. THE CONE-BEAM COVER METHOD

As shown in Figure 1, let $C$ be a spiral defined by

$$C := \left\{ y \in R^3 : y_1 = R\cos(s),\ y_2 = R\sin(s),\ y_3 = s\left(\frac{h}{2\pi}\right),\ s \in I \right\}, \tag{1}$$

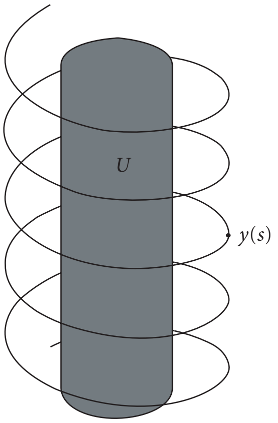

FIGURE 1: The spiral and image space $U$.

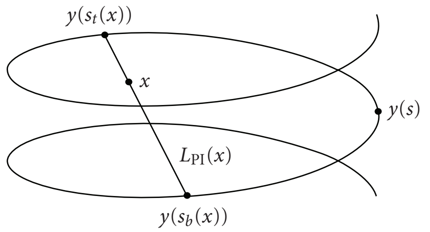

FIGURE 2: PI-line and PI parametric interval. A PI-line is a line segment, the endpoints of which are separated by less than one turn in the spiral. Any $x \in U$ belongs to one and only one PI-line, denoted as $L_{\mathrm{PI}}(x)$. Endpoints of $L_{\mathrm{PI}}(x)$ are denoted as $y(s_b(x))$ and $y(s_t(x))$, where $s_b(x)$ and $s_t(x)$ are angular parameters of the endpoints and $s_b(x) < s_t(x)$. The interval $I_{\mathrm{PI}}(x) := [s_b(x), s_t(x)]$ is called PI parametric interval. The section of the spiral between $y(s_b(x))$ and $y(s_t(x))$ is called PI arc, denoted as $C_{\mathrm{PI}}(x)$.

where $s$ is the angular parameter, $h > 0$ is the spiral pitch and $I := [a, b]$, $b > a$. Let $U$ be the image space defined by

$$U := \left\{ x \in R^3 : x_1^2 + x_2^2 < r^2,\ c\left(\frac{h}{2\pi}\right) < x_3 < d\left(\frac{h}{2\pi}\right) \right\}, \tag{2}$$

where $0 < r < R$, $c < d$, $a = c - s_\Delta$, $b = d + s_\Delta$, $s_\Delta$ is the necessary offset of the angular parameter [1–4]. It is assumed that an object $f(x)$ is centered at the coordinate origin and supported by $U$, that is, $f(x) = 0$ if $x \notin U$. The cone-beam (at vertex $y$) projection of $f$ is defined by

$$D_f(y, \beta) := \int_0^\infty f(y + t\beta)dt, \quad \beta \in S^2. \tag{3}$$

Katsevich's FBP inversion formula [3] can be represented as

$$\begin{aligned} f(x) = -\frac{1}{2\pi^2} \int_{I_{\mathrm{PI}}(x)} \frac{1}{|x - y(s)|} \\ \times \int_0^{2\pi} \frac{\partial}{\partial q} D_f(y(q), \Theta(s, x, y))\Big|_{q=s} \frac{d\gamma}{\sin\gamma} ds, \end{aligned} \tag{4}$$

where $I_{\mathrm{PI}}(x) := [s_b(x), s_t(x)]$ is the PI parametric interval which is determined by the PI line $L_{\mathrm{PI}}(x)$ passing through $x$ [12], see Figure 2.

This formula can be rewritten as

$$f(x) := -\frac{1}{2\pi^2} \int_{I_{\mathrm{PI}}(x)} \frac{1}{|x - y(s)|} \Psi(s, \beta(s, x)) ds, \tag{5}$$

where

$$\beta(s, x) = \frac{x - y(s)}{|x - y(s)|}, \tag{6}$$

$$\Psi(s, \beta) := \int_0^{2\pi} \frac{\partial}{\partial q} D_f(y(q), \cos(\gamma)\beta + \sin(\gamma)e(s, \beta))\Big|_{q=s} \frac{1}{\sin\gamma} d\gamma, \tag{7}$$

for the unit vector $\beta$ along which X-ray emitted from $y(s)$ will reach the Tam-Danielsson window [13, 14], see Figure 3.

Equations (5) and (7) imply that the inversion formula is of the filtered-backprojection type. One first computes the shift-invariant filtering of derivative of cone-beam projection using (7) [3, 15, 16]. Then one performs the backprojection according to (5). Here, for every $x \in U$, the backprojection is performed along PI arc $C_{\mathrm{PI}}(x)$ (the PI line method).

In [1], we introduce the cone-beam cover method to perform backprojection in a way different from the PI line method.

*Definition 1.* Let $W(s_0)$ be the Tam-Danielsson window at source point $y(s_0)$ $(s_0 \in I)$, we call

$$V(s_0) := \{x \in U : \hat{x} \in W(s_0)\} \tag{8}$$

cone-beam cover at source point $y(s_0)$, where $\hat{x}$ is the projection of $x$ onto the detector plane, see Figure 4.

The following theorem on the cone-beam cover was proved in [1]. It is the footstone of the cone-beam cover method.

**Theorem 1.** *For $x \in U$, one has*

$$x \in V(s_0) \quad \textit{iff}\ s_0 \in I_{\mathrm{PI}}(x). \tag{9}$$

Applying Theorem 1 to the discrete form of (5):

$$f(x) = -\frac{1}{2\pi^2} \sum_{s \in I_{\mathrm{PI}}(x)} \frac{\Psi(s, \beta(s, x))\Delta s}{|x - y(s)|}, \tag{10}$$

we obtain the following equation

$$f(x) = -\frac{1}{2\pi^2} \sum_{\{s : x \in V(s)\}} \frac{\Psi(s, \beta(s, x))\Delta s}{|x - y(s)|}. \tag{11}$$

Equation (11) implies that the filtered cone-beam projection at any source point $y(s)$ contributes only to the reconstruction of voxels in $V(s)$. Based on this fact, the cone-beam cover

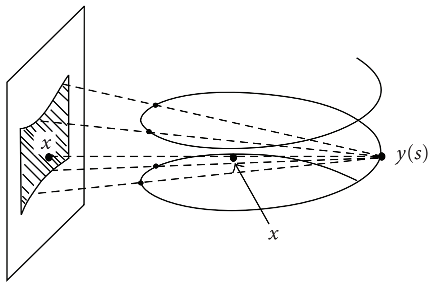

FIGURE 3: Tam-Danielsson window. The Tam-Danielsson window is a region on the detector plane. It is bounded by the cone-beam projection of the upper and lower turns of the spiral onto the detector plane.

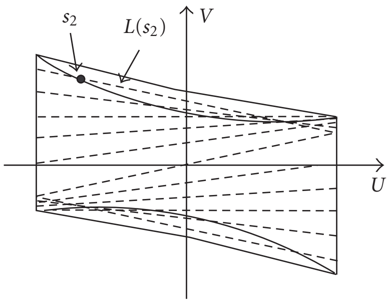

FIGURE 5: The family of lines $L(s_2)$. The filtering is performed along lines $L(s_2)$.

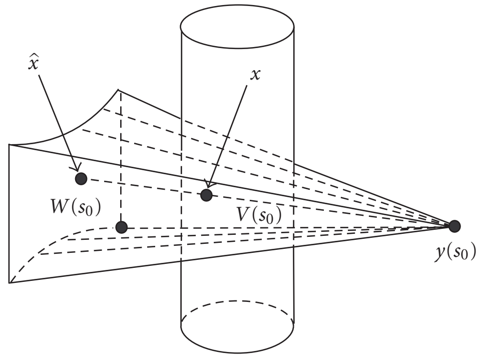

FIGURE 4: Cone-beam cover $V(s_0)$. Let $\widetilde{V}(s_0)$ be the cone with vertex $y(s_0)$ and base $W(s_0)$, then $V(s_0) = \widetilde{V}(s_0) \bigcap U$.

Step 1. for each voxel $x$ of image space, set $f(x) = 0$.
Step 2. for each source point $y(s)$, $s \in I$
do
(1) Filtering of derivative of the cone-beam projection, get $\Psi(s, \beta(s, x))$.
(2) Backprojecting for $x \in V(s)$

$$f(x) = f(x) + \left(-\frac{1}{2\pi^2}\right)\left(\frac{1}{|x - y(s)|}\right) \times \Psi(s, \beta(s, x))\,\Delta s.$$

end

ALGORITHM 1: Sequential implementation of Katsevich's FBP inversion formula.

method provides an approach to performing backprojection. The key point is as follows.

At any source point $y(s)$, the cone-beam projection

$$D_f(y(s), \beta) := \int_0^\infty f(y(s) + t\beta)\,dt \tag{12}$$

is truncated by those unit vector $\beta$ along which X-rays emitted from $y(s)$ will reach a region slightly larger than Tam-Danielsson window $W(s)$ on the detector plane [3]. As shown in Figure 5, the region is bounded by the parallelogram. Dealing with this cone-beam projection, one first computes the shift-invariant filtering of derivative of the cone-beam projection using (7) along the lines $L(S_2)$ (see Figure 5), as discussed elsewhere [3, 16]. Then one performs the backprojection according to (11). Here with the filtered cone-beam projection at source point $y(s)$ the backprojection is performed only for $x \in V(s)$.

Note that using the above strategy, both filtering and backprojecting on a single cone-beam projection can be performed independently. This property of the strategy leads to the sequential and parallel algorithms we will describe later.

A sequential implementation of Katsevich's FBP formula is stated in Algorithm 1. More details and experiment results about this implementation can be found in [1].

### 3. PARALLEL IMPLEMENTATION

We parallelize the above sequential implementation by partitioning the source points set. Let $\bar{I}$ be a discretization of $I$ (angular parameter interval), $p$ be the number of processors. If $\bar{I}$ is partitioned into $p$ subsets

$$\bar{I} = \bigcup_{j=1}^{P} I_j, \tag{13}$$

then a parallel implementation of Katsevich's FBP formula can be pseudocoded Algorithm 2.

In Algorithm 2, each processor $j$ just operates on its assigned cone-beam projections and gets $f_j(x)$, and no communication between different processors is needed during the processing.

### 4. EXPERIMENTS

We do all computations on the Beowulf cluster CCSE-HP at Peking University. The cluster consists of 1 master node, 2 login nodes, 4 I/O nodes, and 128 computing nodes. All the nodes are on a Gigabit Ethernet. The computing nodes are also connected by 4 X InfiniBand (10 Gbps). The system configuration is listed in Table 1.

Table 1: System configuration.

| | |
|---|---|
| Computing node | HP ProLiant DL360 G4 server, Dual 64-bit 3.2 GHz XeonTM DP, 1 MB L2 cache, 4 GB DDR333 RAM, 73 GB Ultra320 SCSI disk, Voltaire HCA 400 PCI-X dual-port 4X InfiniBand (10 Gbps) host channel adapter |
| I/O node | HP ProLiant DL380 G4 server, Dual 64-bit 3.2 GHz XeonTM DP, 1 MB L2 cache, 4 GB DDR333 RAM, Gigabit Ethernet adapter, HP Smart Array 6402/128M SCSI controller |
| Master node | Same as the computing node, except that the RAM is 2 GB |
| Login node | Same as the computing node, except the InfiniBand host interface |
| InfiniBand switch | Voltaire ISR 9288 |
| Gigabit Ethernet switch | HP ProCurve Switch 2848 |
| Storage | HP StorageWorks Modular Smart Array 30, 7TB (RAID5) |
| Operating system | Red Hat EL WS 3.0 |
| Compilers | Intel 64-bit Compiler 9.0.021 (c/c + +) |
| Parallel Environment | mpich 1.2.6-ib |

Step 1. for each voxel $x$ of image space, set $f(x) = 0$.
Step 2. for each processor $j$ $(j = 1, \dots, P)$,
do
for each voxel $x$ of image space, set $f_j(x) = 0$.
for each source point $y(s)$, $s \in I_j$
do
(1) Filtering of derivative of the cone-beam projection, get $\Psi(s, \beta(s, x))$.
(2) Backprojecting for $x \in V(s)$

$$f_j(x) = f_j(x) + \left(-\frac{1}{2\pi^2}\right)\left(\frac{1}{|x - y(s)|}\right) \times \Psi(s, \beta(s, x))\,\Delta s.$$

end
set $f(x) = f(x) + f_j(x)$.
end

Algorithm 2: Parallel implementation of Katsevich's FBP inversion formula.

The parameters of the data collection protocol are given in Table 2.

We use the 3D Shepp-Logan phantom [11] to test our parallel algorithm. The phantom consists of 10 ellipsoids as specified in Table 3.

Figure 6 shows the reconstructed images from sequential and parallel implementations at slices of $x_3 = -0.255$, $x_2 = -0.067$, and $x_1 = -0.067$, respectively. We use the gray scale window $[1.01, 1.03]$ to make low contrast features visible.

In terms of runtime, speedup $S$ is defined as the ratio of the time taken to solve a problem on a single processor to the time required to solve the same problem on a parallel computer with $P$ identical processors. Efficiency $E$ is defined as the ratio of speedup to the number of processors:

Table 2: Parameters of the data collection protocol.

| | |
|---|---|
| $R$ (radius of the spiral) | 3 |
| $h$ (pitch of the spiral) | 1 |
| $r$ (radius of the object) | 1 |
| $[c, d]$ | $[-2\pi, 2\pi]$ |
| $s_\Delta$ | 2.26 |
| $[a, b]$ | $[-2\pi - 2.26, 2\pi + 2.26]$ |
| Sampling number of source points per tun | 600 |
| Detector array size | $100 \times 500$ |
| Image volume | $256 \times 256 \times 256$ |

$$S = \frac{T_1}{T_P}, \qquad E = \frac{S}{P}, \tag{14}$$

where $T_1$ and $T_P$ represent the computing time for one processor and $P$ processors respectively [17]. Figures 7 and 8 display the speedup and efficiency of Algorithm 2, respectively.

### 5. DISCUSSIONS

Since the computation of image reconstruction in Algorithm 2 is mathematically equivalent to that in Algorithm 1, the image reconstructed by Algorithm 2 is the same as that done by Algorithm 1.

Figures 7(a) and 8(a) depict the algorithm's speedup and efficiency with the uniform partition of $\bar{I}$. We can see that the speedup increases from 1.0 to 22.9 while the efficiency decreases from 100.0% to 71.5% when the number of processors varies from 1 to 32. Obviously, the efficiency is not satisfactory with four or more processors. Figure 9 displays

TABLE 3: Parameters of the low-contrast Shepp-Logan phantom.

| No. | $a$ | $b$ | $c$ | $x_{10}$ | $x_{20}$ | $x_{30}$ | $\phi$ | $A$ |
|---|---|---|---|---|---|---|---|---|
| 1 | 0.6900 | 0.920 | 0.900 | 0.00 | 0.000 | 0.000 | 0 | 2.00 |
| 2 | 0.6624 | 0.874 | 0.880 | 0.00 | 0.000 | 0.000 | 0 | −0.98 |
| 3 | 0.4100 | 0.160 | 0.210 | −0.22 | 0.000 | −0.250 | 108 | −0.02 |
| 4 | 0.3100 | 0.110 | 0.220 | 0.22 | 0.000 | −0.250 | 72 | −0.02 |
| 5 | 0.2100 | 0.250 | 0.500 | 0.00 | 0.350 | −0.250 | 0 | 0.02 |
| 6 | 0.0460 | 0.046 | 0.046 | 0.00 | 0.100 | −0.250 | 0 | 0.02 |
| 7 | 0.0460 | 0.023 | 0.020 | −0.08 | −0.650 | −0.250 | 0 | 0.01 |
| 8 | 0.0460 | 0.023 | 0.020 | 0.06 | −0.650 | −0.250 | 90 | 0.01 |
| 9 | 0.0560 | 0.040 | 0.100 | 0.06 | −0.105 | 0.625 | 90 | 0.02 |
| 10 | 0.0560 | 0.056 | 0.100 | 0.00 | 0.100 | 0.625 | 0 | −0.02 |

* $a$, $b$, $c$ are the lengths of half-axes of ellipsoid. $x_{10}$, $x_{20}$, $x_{30}$ are the coordinates of the center. $\phi$ is the rotation angle around $x_3$-axis, $A$ is the incremental density.

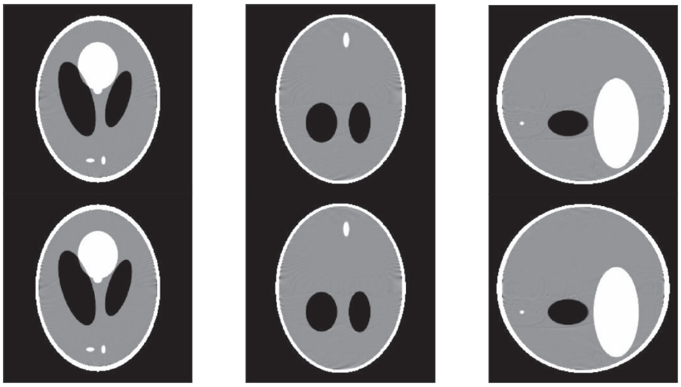

(a) (b) (c)

FIGURE 6: (a) Slice: $x_3 = -0.255$; (b) slice: $x_2 = -0.067$; (c) slice: $x_1 = -0.067$. Top: reconstructed image by Algorithm 1; bottom: reconstructed image by Algorithm 2. Since the computation of image reconstruction in Algorithm 2 is same as that in Algorithm 1, the images reconstructed by Algorithm 2 are same as those by Algorithm 1.

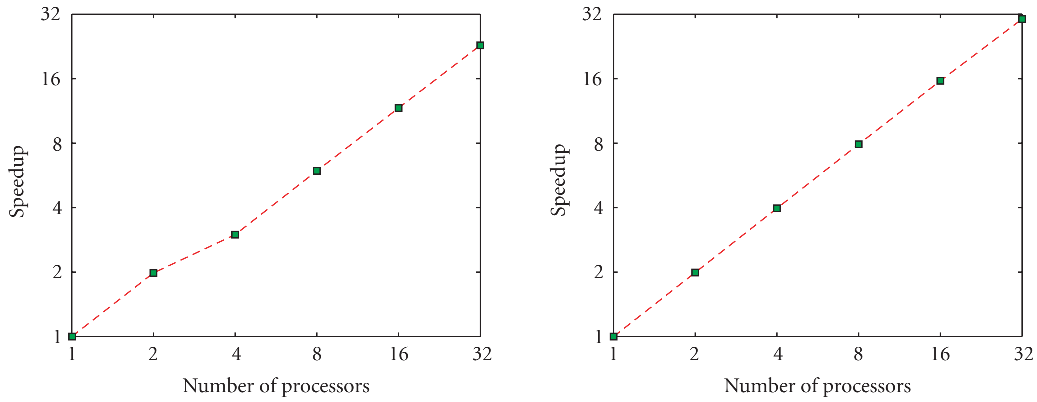

(a) (b)

FIGURE 7: (a) Speedup with the uniform partition of $\overline{I}$; (b) speedup with the nonuniform partition of $\overline{I}$. The speedup with the nonuniform partition of $\overline{I}$ is better than that with the uniform partition of $\overline{I}$.

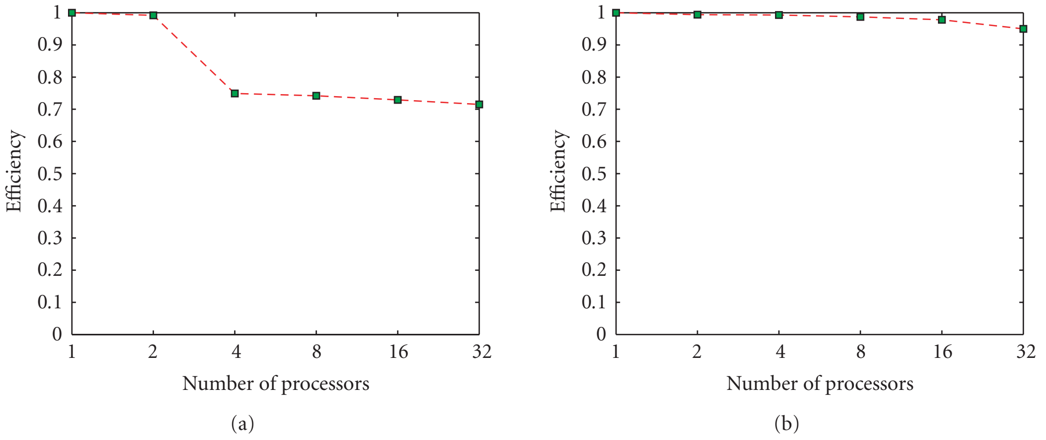

FIGURE 8: (a) Efficiency with the uniform partition of $\overline{I}$; (b) efficiency with the nonuniform partition of $\overline{I}$. The efficiency with nonuniform partition of $\overline{I}$ is better than that with the uniform partition of $\overline{I}$.

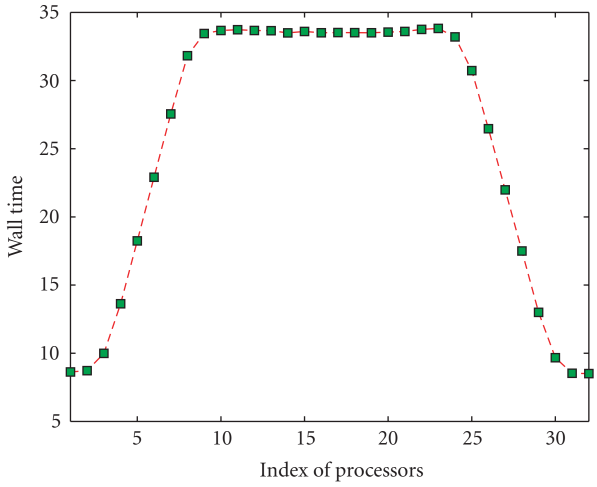

FIGURE 9: Wall time of each processor with the uniform partition of $\overline{I}$. The graph looks like a trapezoid, exhibiting a problem of load imbalance. The processors in the middle bear higher load, while those on the two sides bear lower load.

the wall time[1] for each processor. The graph looks like a trapezoid, exhibiting a problem of load imbalance. The processors in the middle bear higher load, while those on the two sides bear lower load.

The reason is as follows. When the X-ray source point goes near the bottom or top of the image space, the cone-beam cover becomes smaller and smaller, making the load of the corresponding processors lower and lower. Nonuniform partition of $\overline{I}$ can overcome the load imbalance. The following describes a scheme of nonuniform partition of $\overline{I}$.

[1] "Real world" time (what the clock on the wall shows), as opposed to the system clock's idea of time. http://computing-dictionary.thefreedictionary.com/wall%20time.

Recall that $I = [a, b]$ is the angular parameter interval of the spiral, the interval $[c, d]$ expresses the axial position of image space $U$. Let $s_\Delta$ be the maximal axial-direction distance of points in the cone-beam cover from the source point, then $a = c - s_\Delta$, $b = d + s_\Delta$. Figure 10 illustrates computation time for the filtering and backprojecting at source point $y(s)$.

We divide $[a, a + 2s_\Delta)$, $[a + 2s_\Delta, b - 2s_\Delta)$, $[b - 2s_\Delta, b]$ into $p$ uniform subintervals, respectively, as

$$[a, a + 2s_\Delta) = \bigcup_{j=1}^{p} I_{1,j}, \qquad [a + 2s_\Delta, b - 2s_\Delta) = \bigcup_{j=1}^{p} I_{2,j}, \qquad [b - 2s_\Delta, b] = \bigcup_{j=1}^{p} I_{3,j}, \tag{15}$$

and let

$$I_j = I_{1,j} \bigcup I_{2,j} \bigcup I_{3,j}, \quad j = 1, \ldots, p, \tag{16}$$

then

$$[a, b] = \bigcup_{j=1}^{p} I_j \tag{17}$$

determines a nonuniform partition of $\overline{I}$.

Figures 7(b) and 8(b) display the algorithm's speedup and efficiency with the above nonuniform partition of $\overline{I}$. We can see that the speedup increases from 1.0 to 30.4 and the efficiency decreases from 100% to 95% when the number of processors varies from 1 to 32. The speedup and the efficiency with the nonuniform partition of $\overline{I}$ are apparently better than those with the uniform partition of $\overline{I}$. The algorithm's speedup and efficiency maintain a higher level with larger image sizes. The algorithm's speedup and efficiency with the image volume 512 × 512 × 512 are displayed in Figure 11.

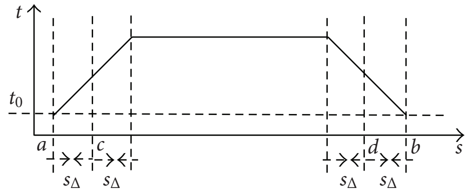

FIGURE 10: Computation time for the filtering and backprojecting at source point $y(s)$. The time of the filtering is same at all source points and denoted by $t_0$.

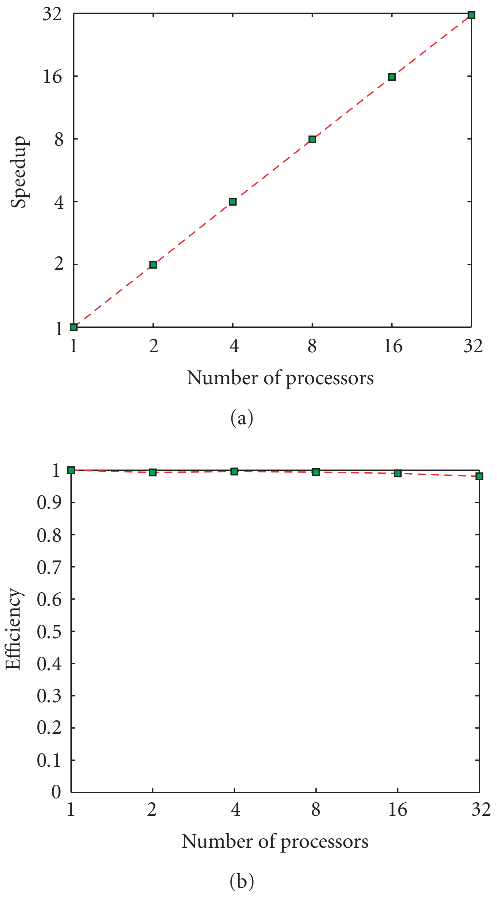

FIGURE 11: The illustration of the algorithm's speedup and efficiency with the image volume $512 \times 512 \times 512$.

The idea of the cone-beam cover is a key point for us to design the parallel implementation of Katsevich's inversion formula. From the perspective of the cone-beam cover, backprojecting any filtered cone-beam projection can be performed independently. Therefore a partition of cone-beam projections gives a parallel implementation of image reconstruction. The presented results demonstrate the high performance of the proposed parallel algorithm. The cone-beam cover method can also be employed to establish parallel schemes for other reconstruction algorithms, such as Feldkamp-type algorithms [11, 18], Zou and Pan's algorithm [19, 20]. In addition, parallel implementation of the Katsevich's FBP algorithm based on PI line method would be an important topic of future research.

### 6. CONCLUSIONS

For spiral cone-beam CT, parallel computing is an effective approach to improving the reconstruction efficiency. Basing on the cone-beam cover method, we have proposed an efficient parallel implementation of Katsevich's FBP algorithm and demonstrated its high performance with numerical simulations. Further work is under active investigation.

### ACKNOWLEDGMENTS

This work is supported in part by the National Basic Research Program of China under Grant 2003CB716101, National Science Foundation of China under Grant 60325101, 60532080, 60372024, the key project of Chinese Ministry of Education under Grant 306017, Engineering Research Institute of Peking University. We would like to thank Professor Linbo Zhang from the State Key Laboratory of Scientific and Engineering Computing, Chinese Academy of Sciences for valuable discussions. We would also like to thank the Center for Computational Science and Engineering of Peking University for providing computing facility.

### REFERENCES

[1] J. Yang, Q. Kong, T. Zhou, and M. Jiang, "Cone-beam cover method: an approach to performing backprojection in Katsevich's exact algorithm for spiral cone-beam CT," *Journal of X-Ray Science and Technology*, vol. 12, pp. 199–214, 2004.

[2] A. Katsevich, "Theoretically exact filtered backprojection-type inversion algorithm for spiral CT," *SIAM Journal on Applied Mathematics*, vol. 62, pp. 2012–2026, 2002.

[3] A. Katsevich, "Improved exact FBP algorithm for spiral CT," *Advances in Applied Mathematics*, vol. 32, pp. 681–697, 2004.

[4] A. Katsevich, "Analysis of an exact inversion algorithm for spiral cone-beam CT," *Physics in Medicine and Biology*, vol. 47, pp. 2583–2597, 2002.

[5] W. L. Nowinski, "Parallel implementation of the convolution method in image reconstruction," in *CONPAR 90-VAPP IV*, H. Elurkhardt, Ed., vol. 457 of *Lecture Notes in Computer Science*, pp. 355–364, Zurich, Switzerland, September 1990.

[6] J. B. T. M. Roerdink and M. A. Westenberg, "Data-parallel tomographic reconstruction: a comparison of filtered backprojection and direct Fourier reconstruction," *Parallel Computing*, vol. 24, pp. 2129–2142, 1998.

[7] C. M. Chen, S. Y. Lee, and Z. H. Cho, "A parallel implementation of 3D CT image reconstruction on hypercube multiprocessor," *IEEE Transactions on Nuclear Science*, vol. 37, no. 3, pp. 1333–1346, 1990.

[8] Z. Cho, C. Chen, and S.-Y. Lee, "Incremental algorithm: a new fast backprojection scheme for parallel beam geometries," *IEEE Transactions on Medical Imaging*, vol. 9, no. 2, pp. 207–217, 1990.

[9] D. Reimann, C. Chaudhary, M. J. Flynn, and I. Sethi, "Cone beam tomography using MPI on heterogeneous workstation clusters," in *Proceedings of the 2nd MPI Developer's Conference*, pp. 142–148, Notre Dame, Ind, USA, 1996.

[10] C. Laurent, F. Peyrin, J. M. Chassery, and M. Amiel, "Parallel image reconstruction on MIMD computers for three-dimensional cone beam tomography," *Parallel Computing*, vol. 24, pp. 1461–1479, 1998.

[11] G. Wang, T. H. Lin, P. C. Cheng, and D. M. Shinozaki, "A general cone-beam reconstruction algorithm," *IEEE Transactions on Medical Imaging*, vol. 12, no. 3, pp. 486–496, 1993.

[12] M. Defrise, F. Noo, and H. Kudo, "A solution to the long-object problem in helical cone-beam tomography," *Physics in Medicine and Biology*, vol. 45, pp. 623–643, 2000.

[13] K. C. Tam, S. Smarasekela, and F. Sauer, "Exact cone-beam CT with a spiral scan," *Physics in Medicine and Biology*, vol. 43, pp. 1015–1024, 1998.

[14] P. E. Danielsson, P. Edholm, and M. Seger, "Towards exact 3D-reconstruction for helical cone-beam scanning of long objects: a new detector arrangement and a new completeness condition," in *Proceedings of International Meeting on Fully Three-Dimensional Image Reconstruction in Radiology and Nuclear Medicine*, D. W. Townsend and P. E. Kinahan, Eds., pp. 141–144, Pittsburgh, Pa, USA, 1997.

[15] F. Noo, J. Pack, and D. Heuscher, "Exact helical reconstruction using native cone-beam geometries," *Physics in Medicine and Biology*, vol. 48, pp. 3787–3818, 2003.

[16] H. Yu and G. Wang, "Studies on implementation of the Katsevich algorithm for spiral cone-beam CT," *Journal of X-Ray Science and Technology*, vol. 12, pp. 97–116, 2004.

[17] A. Grama, G. Karypis, V. Kumar, and A. Gupta, *Introduction to Parallel Computing: Design and Analysis of Algorithms*, Addison Wesley, Reading, Mass, USA, 2nd edition, 2003.

[18] L. A. Feldkamp, L. C. Davis, and J. W. Kress, "Practical cone-beam algorithm," *Journal of the Optical Society of America A: Optics and Image Science, and Vision*, vol. 1, no. 6, pp. 612–619, 1984.

[19] Y. Zou and X. Pan, "Exact image reconstruction on PI-lines from minimum data in helical cone-beam CT," *Physics in Medicine and Biology*, vol. 49, pp. 941–959, 2004.

[20] Y. Zou and X. Pan, "Image reconstruction on PI-lines by use of filtered backprojection in helical cone-beam CT," *Physics in Medicine and Biology*, vol. 49, pp. 2717–2731, 2004.

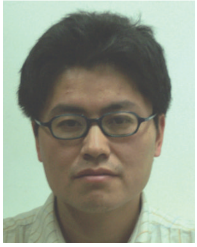

**Jiansheng Yang** is a Lecturer in School of Mathematical Science, Department of Information Science, Peking University. He received his B.S. (1988) and M.S. (1991) degrees in mathematics from Peking University. He obtained his Ph.D. (1994) degree in applied mathematics from Peking University. His research interests include image reconstruction and wavelet.

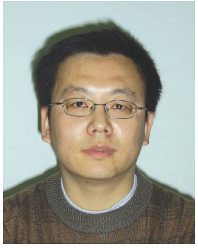

**Xiaohu Guo** is now a Postdoctor in School of Mathematical Science, Department of Information Science, Peking University. He obtained his Ph.D. degree in parallel computing from Institute of Computational Mathematics and Scientific/Engineering Computing of CAS, Beijing, China in 2004, and obtained his B.S. and M.S. degrees in computing mathematics from Department of Mathematics, Ningxia University, China in 1997 and 2000, respectively. His interests are parallel computing, image processing and reconstruction, CFD.

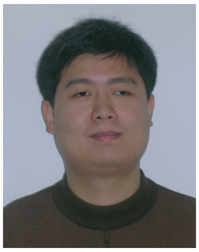

**Qiang Kong** received his B.S. degree in applied mathematics from Peking University, China in 2003. He is currently pursuing his M.S. degree in Peking University. His research interests include image reconstruction and parallel computing.

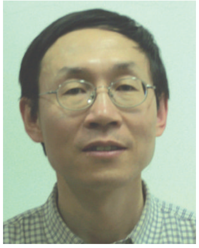

**Tie Zhou** is an Associate Professor of computational mathematics at Peking University. He received his Ph.D. degree in mathematics from Peking University (1994). He received his M.S. degree in mathematics from Peking University in 1990. He received his B.A. degree in mathematics from Peking University in 1985. His research interests include biomedical imaging, numerical partial differential equations, computational fluid dynamics.

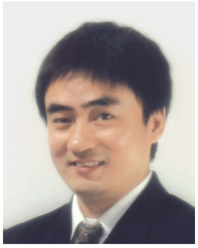

**Ming Jiang** is a Professor with the School of Mathematical Science, Department of Information Science, Peking University. He obtained his B.S. and Ph.D. degrees in nonlinear analysis from Peking University in 1984 and 1989, respectively. He was Associate Professor with Department of Applied Mathematics, Beijing Institute of Technology from 1989 to 1995; Postdoctoral Fellow with Microprocessor Laboratory, International Centre for Theoretical Physics, Italy, from 1996 to 1997; Visiting Professor and Associate Director of CT/Micro-CT Laboratory, Department of Radiology, University of Iowa, USA, from 2000 to 2002. His interests are image reconstruction, restoration, and analysis.

